# Binary photoacoustic tomography for improved vasculature imaging

**DOI:** 10.1117/1.JBO.26.8.086004

**Published:** 2021-08-18

**Authors:** Jaya Prakash, Sandeep Kumar Kalva, Manojit Pramanik, Phaneendra K. Yalavarthy

**Affiliations:** a Indian Institute of Science, Department of Instrumentation and Applied Physics, Bangalore, Karnataka, India; b Nanyang Technological University, School of Chemical and Biomedical Engineering, Singapore, Singapore; c Indian Institute of Science, Department of Computational and Data Sciences, Bangalore, Karnataka, India

**Keywords:** photoacoustic tomography, inverse problems, image segmentation, binary tomography, regularization theory

## Abstract

**Significance:** The proposed binary tomography approach was able to recover the vasculature structures accurately, which could potentially enable the utilization of binary tomography algorithm in scenarios such as therapy monitoring and hemorrhage detection in different organs.

**Aim:** Photoacoustic tomography (PAT) involves reconstruction of vascular networks having direct implications in cancer research, cardiovascular studies, and neuroimaging. Various methods have been proposed for recovering vascular networks in photoacoustic imaging; however, most methods are two-step (image reconstruction and image segmentation) in nature. We propose a binary PAT approach wherein direct reconstruction of vascular network from the acquired photoacoustic sinogram data is plausible.

**Approach:** Binary tomography approach relies on solving a dual-optimization problem to reconstruct images with every pixel resulting in a binary outcome (i.e., either background or the absorber). Further, the binary tomography approach was compared against backprojection, Tikhonov regularization, and sparse recovery-based schemes.

**Results:** Numerical simulations, physical phantom experiment, and *in-vivo* rat brain vasculature data were used to compare the performance of different algorithms. The results indicate that the binary tomography approach improved the vasculature recovery by 10% using *in-silico* data with respect to the Dice similarity coefficient against the other reconstruction methods.

**Conclusion:** The proposed algorithm demonstrates superior vasculature recovery with limited data both visually and based on quantitative image metrics.

## 1 Introduction

Photoacoustic tomography (PAT) is an imaging technique that enables us to visualize vasculature networks at high resolution at depths much greater than optical microscopy.^1^^,^^2^ PAT can also resolve tissue chromophores such as oxyhemoglobin and deoxyhemoglobin, which allows estimation of tissue oxygentation levels.^1^^,^^3^ Accurate estimation of tissue oxygentation is very important in the fields of neuroimaging, cancer diagnostics, and critical care medicine.^4^^,^^5^ PAT involves collection of broadband acoustic waves generated by thermoelastic expansion of tissue upon absorption of light (illuminated at near-infrared wavelengths).^6^^,^^7^ PAT image reconstruction involves recovery of the acoustic source distribution from the detected broadband acoustic waves, which can be performed using analytical or model-based methods.^8^ Analytical methods are computationally less expensive compared to model-based image reconstruction.^8^^,^^9^ In contrary, model-based schemes are known to generate accurate images compared to analytical schemes, specifically in scenarios where irregular acquisition geometry is involved or limited data is acquired.^9^^,^^10^ Even with the model-based schemes, PAT inversion becomes difficult when the number of ultrasound detector positions is limited, and the situation gets aggravated due to the presence of noise in the data. More recently deep learning-based image postprocessing schemes have been developed for artifact removal and for limited data situations in PAT/microscopy;^11^^–^^13^ however, these schemes were trained on simulated data and generating large experimental data is very difficult.

Segmentation approaches were proposed to delineate skin lining in mesoscopic photoacoustic images.^14^ Active contours-based image segmentation was used to obtain the PAT region of interest (ROI), the obtained ROI was then used to perform fluence correction to make PAT more quantitative.^15^^,^^16^ Different computational techniques have been reported in the literature to obtain vascular network in photoacoustic imaging. Vesselness filter has been proposed to enable accurate recovery of vasculature in PAT;^17^^–^^19^ however, questions have been raised on the accuracy of using the vesselness filter (due to generation of artificial vasculature).^20^ Probabilistic approach involving different steps such as smoothing and filtering the data, clustering, vessel-segmentation using clusters, and morphological filling was developed for segmenting vessels in photoacoustic images.^21^ More recently convolution neural network-based approaches were also developed for jointly performing the image reconstruction and segmentation.^22^ Further, deep learning-based approach was proposed for segmenting animal boundary using multi-modal photoacoustic and ultrasound data.^23^ Further, deep learning-based method has also been developed for animal brain imaging.^24^

Notably segmenting vasculature is very important in the context of diagnosis and surgical guidance, specifically for cardiovascular diseases.^25^^,^^26^ In this work, we developed a binary tomography approach wherein we assume the reconstruction distribution contains only two unknowns namely the background and the absorbers. The major contributions of this work include: (a) application of binary tomographic methods for photoacoustic tomographic imaging involving spherical wave propagation as opposed to straight line approximation applied in x-ray computed tomography; (b) showing the utility of binary tomographic methods for improving photoacoustic tomographic imaging of vasculature; and (c) establishing the binary tomographic method superiority in photoacoustic tomographic imaging for providing approximate priors to improve model-based image reconstruction.

Binary tomography problem is solved based on a dual optimization approach with an auxiliary variable posed as a constrained optimization problem. We compared the binary tomography algorithm against standard backprojection approach and model-based reconstruction schemes such as Tikhonov regularization and sparsity-based regularization.^27^ The performance of these different algorithms was validated with *in-silico* data, physical phantom, and *in-vivo* brain vasculature imaging data.

## 2 Methods

### 2.1 Acoustic Forward Problem

The acoustic forward model in PAT can be written as

Ax=b, (1)

where A is the system matrix constructed as explained in Refs. 9 and 28, b indicates the acoustic time series data measured at the detector locations, and x is the initial value of pressure rise at each pixel of the photoacoustic image. Note that the size of the matrix A is NM×NP (NM indicates the times series data at different transducer locations and NP indicates the total number of pixels).

Backprojection is the simplest approach to reconstruct the photoacoustic image from the measured acoustic data and is mathematically given as $x_{\mathrm{bp}}=A^{T}b$.^29^ The reconstructed photoacoustic image quality is often very less using the backprojection approach ($x_{\mathrm{bp}}$) especially when limited data is available.

### 2.2 Model-Based Reconstructions

#### 2.2.1 L2-Norm-based (Tikhonov) regularization

The Tikhonov regularization method has been used by many researchers in the context of photoacoustic imaging. Tikhonov method generates smoother solutions, and the characteristics of the solution are governed by the choice of the regularization parameter. The Tikhonov regularization approach minimizes the following objective function:^30^

$$\Omega_{\mathrm{Tikh}}=\underset{x}{\min}({\left\|Ax-b\right\|}_{2}^{2}+\lambda {\left\|x\right\|}_{2}^{2}),$$ (2)

where the regularization parameter is indicated by λ, which trades-off between the residue of the linear equations and L2-norm of (x). Smoother photoacoustic images could be obtained using higher regularization parameter value. In contrary, a smaller value of λ will increase the noise/high-frequency information in the reconstructed photoacoustic image. Minimization of the objective function ($\Omega_{\mathrm{Tikh}}$) can be performed using normal equations, which results in:^9^^,^^30^

$$x_{\text{Tikh}}={\left(A^{T}A+\lambda I\right)}^{-1}A^{T}b.$$ (3)

Further, since the system matrix is having large dimension (i.e., 51,200×40,000), a least squares QR-based dimensionality reduction was used to perform this inversion.^9^^,^^31^ The regularization parameters used in the Tikhonov regularization approach was chosen heuristically to result in the best possible figure of merit.

#### 2.2.2 L1-Norm-based (sparsity) regularization

Photoacoustic imaging is widely used for cardiovascular imaging due to its ability to image vasculature networks, hence it is reasonable to assume that PA images are sparse in nature. Therefore, it is expected that using non-smooth regularizers, such as the L1-norm-based approach could reconstruct accurate photoacoustic images. Many numerical optimization techniques were proposed for performing sparse recovery-based reconstruction.^9^^,^^32^^,^^33^ The L1-norm-based objective function is given by^33^^–^^35^

$$\Omega_{\ell_{1}}=\underset{x}{\min}({\left\|Ax-b\right\|}_{2}^{2}+\lambda {\left\|x\right\|}_{1}),$$ (4)

where ${\left\|.\right\|}_{1}$ represents the L1-norm. A majorization minimization framework was utilized to solve the L1-norm-based minimization in PAT, as explained in Refs. 32 and 35. The reconstruction parameters used in the majorization minimization approach were chosen heuristically to result in the best possible figure of merit.

### 2.3 Binary Tomography

Binary tomography tries to reconstruct imaging volumes having binary distribution, i.e., $x\in (u_{0},0,u_{1})$ with few number of detection positions.^36^ Performing reconstruction with limited data is particularly essential in the context of PAT, which has direct implication in terms of data-acquisition time or system cost. Binary tomography will try to directly estimate the segmented output from the acquired photoacoustic data. The binary tomography problem is defined as^36^

$$x^{*}=\underset{x}{\mathrm{argmin}}[\inf_{\Phi }(\frac{1}{2}{\left\|Ax-b\right\|}^{2})]\;\text{subjected to }x=\mathrm{sgn}(\Phi ),$$ (5)

where sgn(.) denotes element wise signum function [with sgn(0)=0] and Φ is the auxiliary variable. As can be seen, the above minimization problem is trying to estimate the solution that will have binary distribution based on the sign of the auxiliary variable. The Lagrangian for the above problem is given as^36^

$$L(x,\Phi ,v)=\frac{1}{2}{\left\|Ax-b\right\|}^{2}+v^{T}(x-\mathrm{sgn}(\Phi )).$$ (6)

The variable v is the Lagrangian multiplier associated with equality constraint, i.e., x=sgn(Φ). The above optimization can be solved using a dual optimization approach:

$$v^{*}={\arg\;\max}_{v}[g(v)],$$ (7)

where g(v) is the dual function, which is given as

$$g(v)=\inf_{\Phi ,x}[L(x,\Phi ,v)].$$ (8)

The dual problem in the case of binary tomography (with $u_{0}<u_{1}$) comes out to be

$$v^{*}={\arg\;\min}_{v\in R^{N}}[\frac{1}{2}{\left\|v-A^{T}b\right\|}_{\left(A^{T}A\right)}+p(v)],$$ (9)

where $p(v)=\sum_{i}(|u_{0}|\max(-v_{i},0)+|u_{1}|\max(v_{i},0))$ is an asymmetric one-norm. The primal solution is given as $x^{*}=u_{0}+(u_{1}-u_{0})H(v^{*})$, with H(.) being a heavy-side function. This dual problem is solved using proximal gradient descent. The proximal operator for the binary tomography problem (the above given asymmetric one-norm function) is given by asymmetric soft-thresholding operation, which is defined as

$$S_{a<b}(t)=t-|b|\;\forall \;t\geq |b|\;S_{a<b}(t)=t+|a|\;\forall \;t\leq -|a|\;S_{a<b}(t)=0\;-|a|<t<|b|.$$ (10)

It is important to note that the binary tomography approach is trying to assign a class to the solution based on the sign of the auxiliary variable, hence a direct segmented output will be obtained from the acquired acoustic time series data. This will in essence avoid a two-step approach of reconstructing the image and then performing image segmentation.

### 2.4 Figure of Merit

The proposed scheme was evaluated using a Dice similarity coefficient, which is used to measure the similarity between two sets of data. Let $x_{\text{true}}$ be the ground truth and $x_{\text{recon}}$ be the reconstructed image and then the Dice similarity coefficient is computed as

$$\mathrm{DC}=\frac{2|x_{\text{true}}\cap x_{\text{recon}}|}{\left|x_{\text{true}}|+|x_{\text{recon}}\right|},$$ (11)

where |.| indicates the number of elements (cardinality) in the image.

### 2.5 Numerical Simulations and Experiments

The different methods explained here were assessed with two numerical phantoms namely the Derenzo phantom (to understand the ability to recover objects of different sizes at different locations in the imaging domain) and a blood vessel phantom (as the main aim of PAT is to recover vasculature structures). The photoacoustic pressure intensity was considered as 1 kPa and speed of sound was considered to be homogeneous and constant at 1500  m/s. Note that numerical simulations are used to evaluate the different algorithms as it is difficult to compare the capabilities of different algorithms with realistic *in-vivo* cases due to unknown ground truth.

A two-dimensional imaging region of size 20.1×20.1  mm (corresponding to 402×402  pixels) was considered, and this imaging region housed the Derenzo phantom and the numerical blood vessel phantom. A larger computational grid having of size 50.1×50.1  mm (corresponding to 1002×1002  pixels) was considered for propagating the acoustic wave fields (forward model) and detecting the pressure signals by transducer. A total of 60 or 80 detectors (transducer positions) were placed on the circle of radius 22 mm. The detected simulated data were then added with additive white Gaussian noise to result in an SNR of 30, 40, and 60 dB in the *in-silico* data. Further, the transducer was modeled to have a center frequency of 2.25 MHz (with bandwidth of 70%), resulting in maximum detectable frequency as 3.0375 MHz. A total of about 512 samples were acquired with a sampling period of 50 ns (simulating a typical data acquisition (DAQ) sampling rate used in experiments). The *in-silico* data were then used to reconstruct the photoacoustic image using the different algorithms explained in Secs. 2.1–2.3. Note that the forward model was run on a finer grid, i.e., 20.1×20.1  mm (corresponding to 402×402  pixels), whereas the reconstruction was performed on a coarser domain, i.e., 20.1×20.1  mm (corresponding to 201×201  pixels) to avoid inverse crime. The dimension of the model matrix in the case of 60 detectors was 30,720×40,401 (corresponding to 40,401: 201×201 reconstruction grid and 30,720: 60 detector position each acquiring 512 time samples), and for the case of 80 detectors is 40,960×40,401 (corresponding to 40,401: 201×201 reconstruction grid and 40,960: 80 detector position each acquiring 512 time samples). The speed of sound in all these cases was considered to be constant at 1500  m/s.

Next, these different algorithms were evaluated using experimental photoacoustic data. Initially, a horse-hair phantom was built. This phantom was arranged in a triangular shape, with side-length and diameter of hair measured to be 10 and 0.15 mm, respectively, these three hairs were glued to the pipette tips adhered on acrylic slab. Photoacoustic signals from the horse-hair phantom data were acquired using the experimental setup shown in Fig. 2 of Ref. 37. The phantom was illuminated with a Q-switched Nd:YAG laser operating at 532 nm having a pulse width of 5 ns with 10 Hz repetition rate. The nanosecond laser pulses were delivered on the sample using Four right-angle uncoated prisms (PS911, Thorlabs) and one uncoated plano-concave lens (LC1715, Thorlabs). Note that the light fluence at the sample was about $9\;\mathrm{mJ}/{\mathrm{cm}}^{2}$. The laser and the DAQ systems were synchronized using a sync signal from the laser. An ultrasound transducer having 13 mm diameter active area and about 70% nominal bandwidth (with center frequency of 2.25 MHz) was rotated around the sample (over 360 deg) for recording the photoacoustic signals. The generated PA signals due to photoacoustic effect were first amplified and then filtered using a pulse amplifier (Olympus-NDT, 5072PR). The amplified signals were then recorded using a DAQ card (GaGe, compuscope 4227) with a sampling frequency of 25 MHz. The experimental data were reconstructed using a model matrix with a size of 51,200×40,000 (corresponding to 40,000: 200×200 reconstruction grid and 51,200: 100 detector position each acquiring 512 time samples). *In-vivo* rat brain imaging data were collected using the same experimental setup.^37^ These *in-vivo* data were used to compare the reconstruction performance of different algorithms. The reconstructions presented in this work were implemented on a system with Intel i9-9920X processor with 3.50 GHz, 288 cores, and 128 GB memory.

## 3 Results and Discussions

**Fig. 1:**
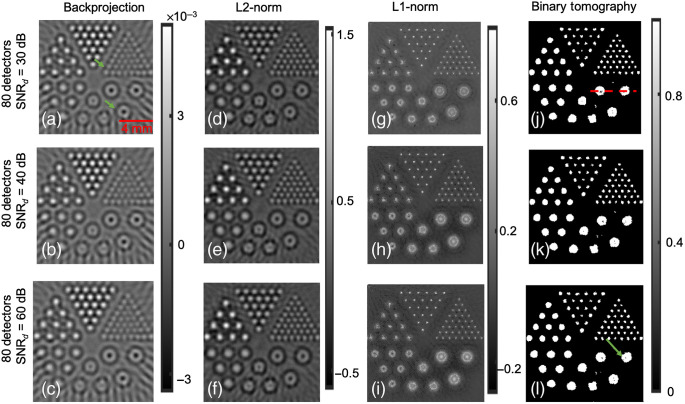
Reconstruction results using Derenzo phantom and 80 detector positions using (a)–(c) backprojection approach and ${\mathrm{SNR}}_{d}=30$, 40, and 60 dB, respectively; (d)–(f) Tikhonov regularization approach and ${\mathrm{SNR}}_{d}=30$, 40, and 60 dB, respectively; (g)–(i) L1-norm approach and ${\mathrm{SNR}}_{d}=30$, 40, and 60 dB, respectively; and (j)–(l) binary tomography approach and ${\mathrm{SNR}}_{d}=30$, 40, and 60 dB, respectively.

Figure 1 shows the reconstructed images obtained using the different algorithms with the Derenzo phantom and 80 detector position. Figures 1(a)–1(c) indicate the reconstruction results corresponding to the backprojection approach with 30, 40, and 60 dB signal-to-noise ratio in data (${\mathrm{SNR}}_{d}$). The reconstruction results corresponding to the L2-norm-based reconstruction for different ${\mathrm{SNR}}_{d}$ (30, 40, and 60 dB) are shown in Figs. 1(d)–1(f). As expected, the reconstructions obtained using the L2-norm approach are more accurate compared to the backprojection algorithm, particularly in reducing the streak artifacts. The L1-norm-based reconstruction outputs are shown in Figs. 1(g)–1(i) for different ${\mathrm{SNR}}_{d}$ values, and it can be seen that the L1-norm-based reconstruction shows improved resolution characteristics compared to L2-norm-based reconstruction. Figures 1(j)–1(l) show the reconstruction corresponding to the binary tomography approach with different levels of noise in the data, as can be seen the reconstruction outputs using the binary tomography approach is able to localize the objects of different sizes with reduced blur. Further, the contrast using the proposed approach is found to be higher compared to other reconstruction schemes.

**Fig. 2:**
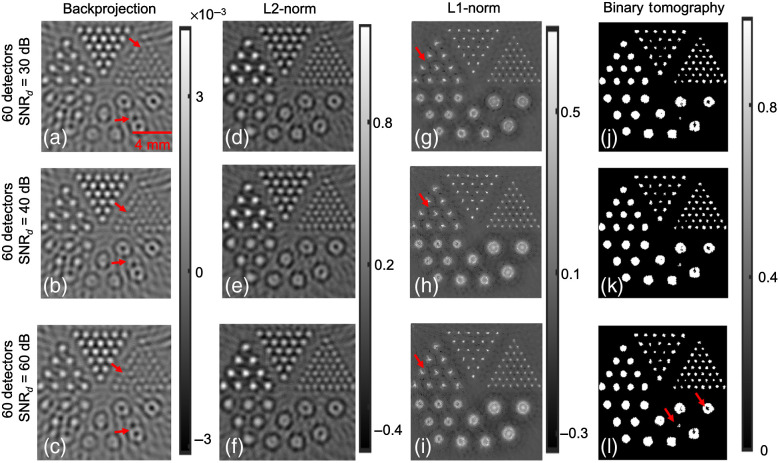
Similar effort as Fig. 1, with 60 acoustic detector positions.

**Fig. 3:**
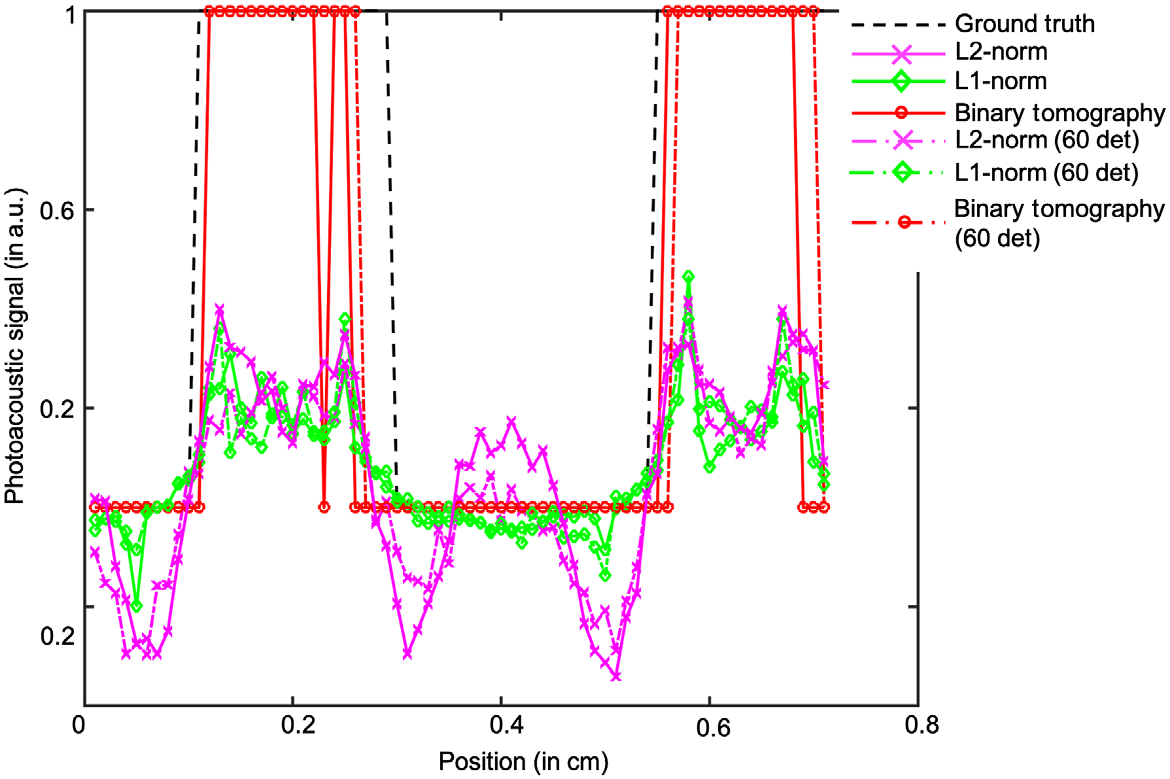
The line plot comparison for the different algorithms (L2-norm, L1-norm, and binary tomography cases) with ${\mathrm{SNR}}_{d}=30\;\mathrm{dB}$ and having 80 and 60 detectors.

Next numerical experiments were performed by reducing the number of detector positions to 60. Figure 2 shows the reconstructed images obtained using with the Derenzo phantom and 60 detector position. Figures 2(a)–2(c) show the reconstruction results obtained using the backprojection algorithm with ${\mathrm{SNR}}_{d}$ as 30, 40, and 60 dB, respectively. In comparison with 80 detector locations, the smaller absorbers were not resolved accurately while using only 60 detector location [as shown with red arrows in Figs. 2(a)–2(c) and green arrows in Fig. 1(a)]. However, these smaller absorbers were accurately resolved using other methods with 60 detector positions (as can be seen in Fig. 2). The reconstruction results corresponding to the Tikhonov regularization approach for different ${\mathrm{SNR}}_{d}$ (30, 40, and 60 dB) is shown in Figs. 2(d)–2(f). The results indicate that the Tikhonov approach was successful in resolving smaller absorbers compared to backprojection approach. The L1-norm regularization-based reconstruction images are shown in Figs. 2(g)–2(i) for different ${\mathrm{SNR}}_{d}$ values. As can be seen with red arrows in Figs. 2(g)–2(i), there are few locations in the imaging domain with large negative values resembling artifacts.^16^^,^^38^ Figures 2(j)–2(l) show the reconstruction results corresponding to the binary tomography approach with different levels of noise in the data, as can be seen the reconstruction outputs using the binary tomography approach has reduced blur and is able to localize the objects of different sizes. Further, the obtained contrast using the proposed approach seems to better compare to other reconstruction schemes. However, it can be seen that spurious artifacts do arise using the proposed approach, and the larger circular absorbers seem to have donut shape [as can be seen with red arrows in Fig. 2(l)]. The line profile along the dashed red line shown in Fig. 1(j) for ${\mathrm{SNR}}_{d}=30\;\mathrm{dB}$ and having 80 and 60 detectors is given in Fig. 3. Figure 3 indicates that the proposed method is able to accurately recover the bigger circles compared to other reconstruction methods. Note that the line-profile with binary tomography and other reconstructions is having smaller size compared to the ground truth. This might be due to lesser number of detector positions, limited bandwidth of the transducer, and the choice of threshold being considered during reconstruction.

**Fig. 4:**
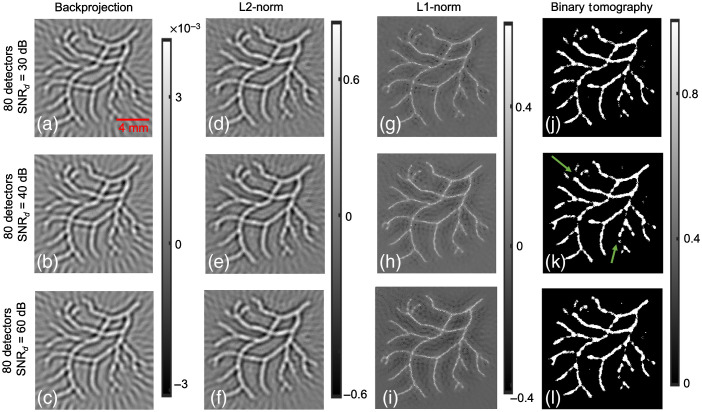
Same effort as Fig. 1 with 80 detectors and blood vessel phantom.

Another numerical experiment using blood vasculature structure was taken up next. Figure 4 shows the results pertaining to blood vasculature phantom and 80 detector locations. Figures 4(a)–4(c) show the vasculature distribution using the backprojection approach at different ${\mathrm{SNR}}_{d}$ values. Figures 4(d)–4(f) indicate the reconstruction distribution obtained using L2-norm regularization with ${\mathrm{SNR}}_{d}$ values being 30, 40, and 60 dB, respectively. The L1-norm regularization-based reconstruction at ${\mathrm{SNR}}_{d}=30$, 40, and 60 dB is shown in Figs. 4(g)–4(i), respectively. As in earlier cases, the L2-norm-based approach seems to have lesser artifacts compared to backprojection approach; L1-norm regularization seems to have better resolution characteristics compared to L2-norm regularization, which is in accordance with earlier works.^33^^,^^35^ Note that L1-norm reconstruction produces reconstructions with improved resolution compared to L2-norm reconstruction due to the presence of soft-thresholding operation during L1-norm minimization. Finally, Figs. 4(j)–4(l) show the reconstruction results corresponding to the binary tomography algorithm. Overall, Fig. 4 indicates that the binary tomography approach is able to recover the vasculature distribution. However, the binary tomography approach seems to present few discontinuities while recovering the vasculature like distribution [shown by green arrows in Fig. 4(k)]. Finally, Figs. 1–4 collectively indicate that binary tomography approach holds potential while recovering structures with binary contrast.

The Dice similarity scores were also computed for the different *in-silico* cases demonstrated in Figs. 1–4, and the same has been shown in Table 1. The Dice similarity scores indicate that the proposed binary tomography approach seems to generate the most accurate representation to the ground truth. For the cases of backprojection, Tikhonov regularization, and L1-based regularization, the reconstructed output was first made non-negative by thresholding with 0, and then segmentation was performed by choosing the mean value as the segmentation threshold.

**Table 1:** Dice similarity scores for the different methods shown in Figs. 1–4. The best results are shown in bold.

Phantom (data noise)	Backprojection	Tikhonov regularization	L1-based regularization	Binary tomography
Fig. 1 (30 dB)	0.8575	0.9002	0.9553	**0.9752**
Fig. 1 (40 dB)	0.8579	0.9013	0.9576	**0.9753**
Fig. 1 (60 dB)	0.8583	0.9012	0.9590	**0.9756**
Fig. 2 (30 dB)	0.8558	0.8859	0.9560	**0.9740**
Fig. 2 (40 dB)	0.8559	0.8863	0.9600	**0.9743**
Fig. 2 (60 dB)	0.8562	0.8863	0.9609	**0.9742**
Fig. 4 (30 dB)	0.8485	0.8903	0.9401	**0.9697**
Fig. 4 (40 dB)	0.8483	0.8932	0.9464	**0.9704**
Fig. 4 (60 dB)	0.8487	0.8929	0.9465	**0.9705**

**Fig. 5:**
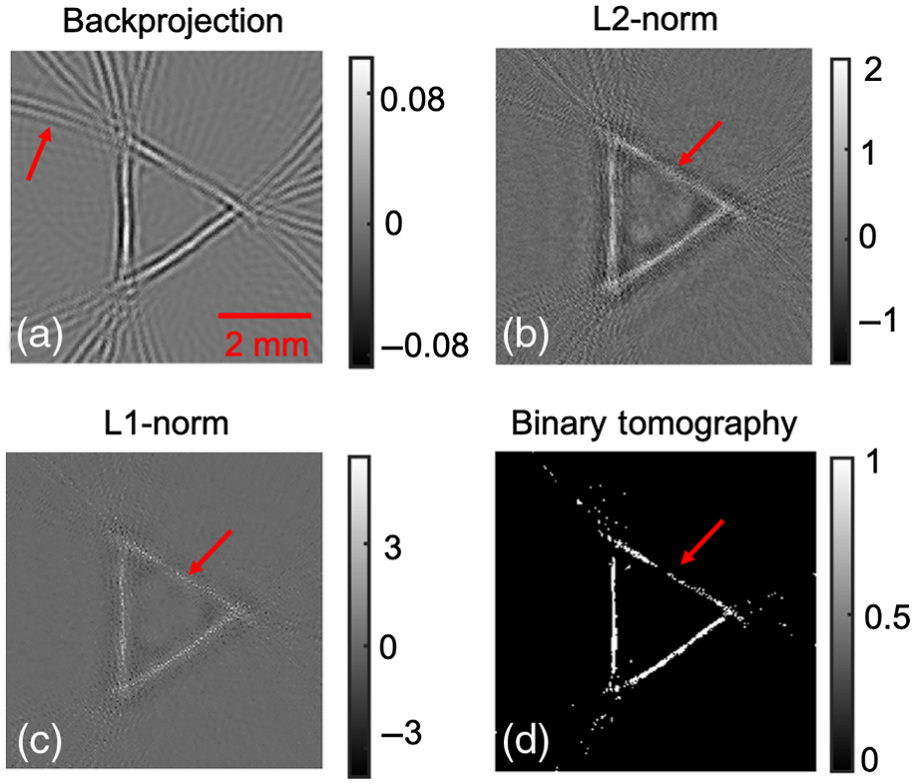
Reconstruction results corresponding to horse hair phantom experiment using (a) backprojection, (b) Tikhonov regularization; (c) L1-norm regularization, and (d) binary tomography.

**Fig. 6:**
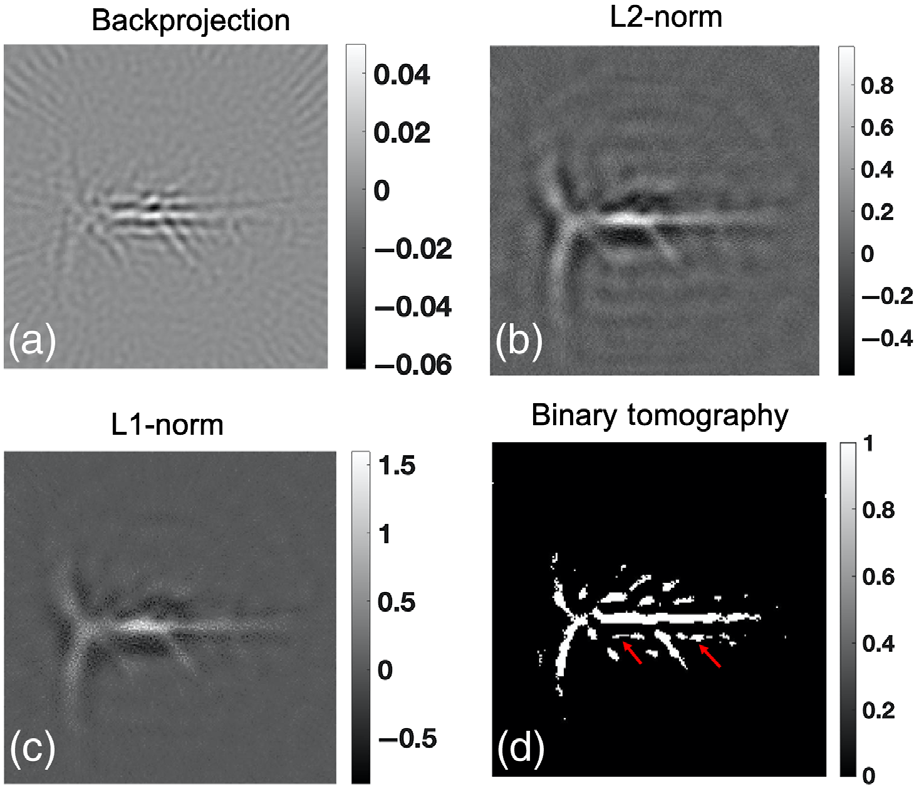
Reconstruction results corresponding to rat brain imaging experiment using (a) backprojection, (b) Tikhonov regularization, (c) L1-norm regularization, and (d) binary tomography.

Next, the binary tomography approach was evaluated using experimental data. Initially, horse-hair physical phantom data were reconstructed using the backprojection, L2-norm regularization, L1-norm regularization, and binary tomography approaches. The results corresponding to the same is shown in Fig. 5. Similar to numerical simulations, the L2-norm regularization scheme [Fig. 5(b)] is found to reconstruct images devoid of artifacts compared to backprojection scheme [shown by red arrow in Fig. 5(a)]. Similarly, the L1-norm regularization-based reconstruction seems to be having increased resolution at cost of amplified noise [shown by red arrow in Fig. 5(c)]. The binary tomography approach is able to generate accurate reconstruction of the horse-hair phantom; however, a discontinuity is observed on one of the hair [same is the case with other methods too; shown by red arrows in Figs. 5(b)–5(d)]. Finally, the different algorithms explained in Sec. 2 were used to reconstruct the *in-vivo* rat brain vasculature distribution. The reconstruction of the vasculature in the rat brain using backprojection approach is shown in Fig. 6(a). The L2-norm and L1-norm-based regularized reconstruction is shown in Figs 6(b) and 6(c), respectively. Figure 6 indicates that L2-norm and L1-norm-based regularized reconstruction seems to outperform the backprojection type reconstruction. Finally, Fig. 6(d) shows the reconstruction results pertaining to the binary tomographic approach. It is evident from Fig. 6 that binary tomography is able to recover larger vasculature; however, the noise and spurious structures seem to appear using the binary tomography approach [double-edge-like structure shown by red arrows in Fig. 6(d)]. Note that it is difficult to compare the reconstructed outputs for experimental data cases using quantitative metrics due to the unavailability of ground truth. Further, metrics such as signal-to-noise ratio and peak-signal-to-noise ratio might heavily bias the comparison toward the binary tomography approach [as background is suppressed (see Figs. 5 and 6)], therefore, we have avoided reporting the quantitative metrics.

**Fig. 7:**
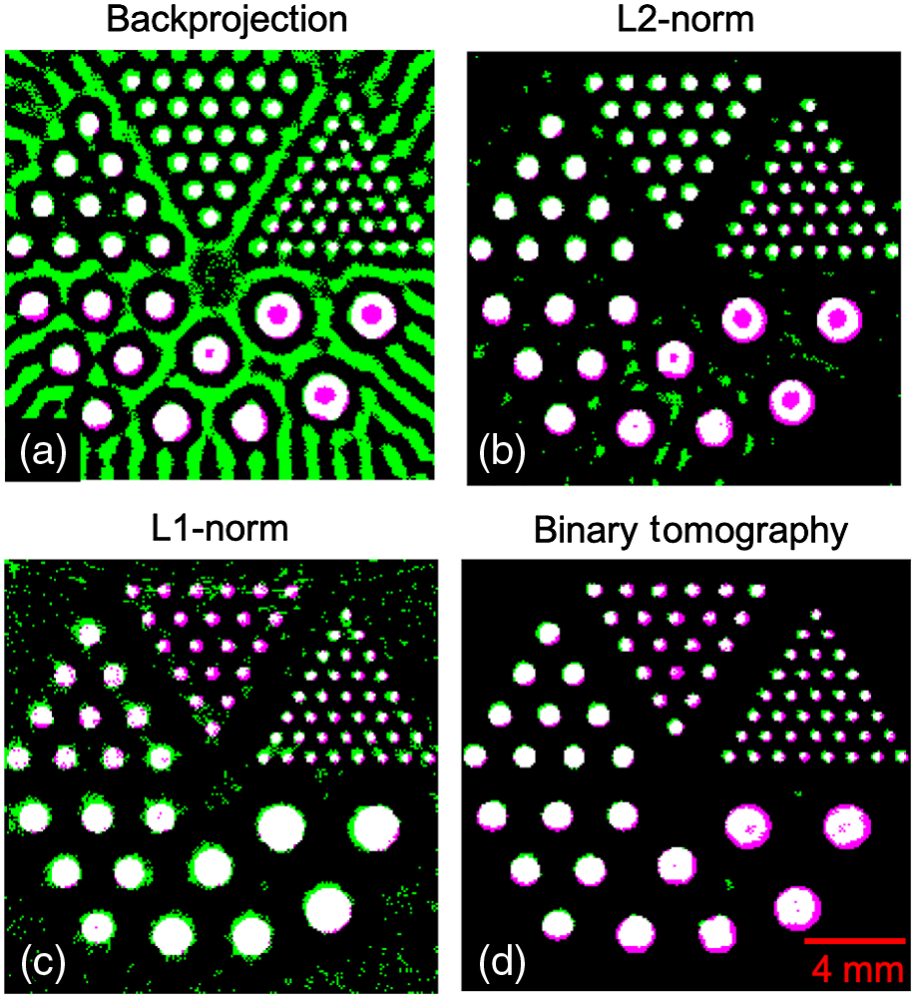
Segmentation comparison with the ground truth using (a) backprojection, (b) Tikhonov regularization, (c) L1-norm regularization, and (d) binary tomography. Here, green color indicates the false positives, and pink color indicates the false negatives obtained using the different algorithms.

To demonstrate the visual comparison of different methods, the segmented results obtained using the backprojection, Tikhonov regularization, and L1-norm regularization are shown in Figs. 7(a)–7(c), respectively. The reconstructions were performed with 80 detector positions and ${\mathrm{SNR}}_{d}=30\;\mathrm{dB}$. Note that the image segmentation was performed using a k-means clustering algorithm having two classes. The images shown here are the image pairs compared to the ground truth, wherein the green color indicates the false positive outcomes and pink color indicates the false negative outcomes. The results corresponding to the binary tomography approach are shown in Fig. 7(d). Note that there are very few artifacts observed using the binary tomography approach compared to the other standard reconstruction schemes.

The results indicate that the proposed binary tomography approach is able to directly reconstruct and delineate the absorbers from the experimental projections; this avoids the two-step approaches (of performing image segmentation after reconstruction). The results also show the appearance of artifacts with the binary tomography approach. However, the binary tomography scheme could be used to obtain the approximate distribution, which can be used as initial seed points for advanced segmentation approaches. Note that the reconstructions obtained using the binary tomography scheme provide only two classes (background and the absorber), making this algorithm to be useful in identifying the vasculature networks. Application like quantification of venous sinus distention for the diagnosis of intracranial hypotension relies more on accurate identification of the size of vasculature than the actual signal strength.^39^ However, in the case of multiscale vasculature, i.e., big arteries/veins to smaller capillaries, this problem can be posed as multiple classes and the binary tomography scheme could fail. In situations of multiple class problems, we could use a discrete tomographic reconstruction approach, which will be taken up as future work. PAT involves reconstruction of the initial pressure rise distribution, which directly relates to the absorbed energy density; however, the emphasis of binary tomography is to recover the vasculature structures accurately, thereby enabling its utility in scenarios such as therapy monitoring and hemorrhage detection in different organs.

The reconstruction times for the Tikhonov, L1-norm, and binary tomography reconstructions are 75.12, 1968.83, and 15.68 s, respectively. Further, the L1-norm-based reconstruction tends to be more noisy, as the L1-norm is not defined close to zero and the derivative of the L1-norm will be an approximation thereby amplifying the noise. The regularization parameters were chosen to result in the best possible reconstruction using the Tikhonov, L1-norm, and binary tomography methods. The regularization parameter does have an influence on the image quality, and the same was modeled and removed using a two-step approach as explained in Refs. 9 and 40. However, in this work, we have chosen the value optimally to result in the best possible figure of merit and avoid biasing the comparison. Notably the quantitative results obtained (in terms of Dice similarity scores) using the binary tomography approach is the best among the different methods with an advantage of being single step scheme. The proposed approach directly works with the acquired photoacoustic data (being robust at different noise levels as found in simulations) and hence does not require large training data to perform image segmentation using learning methods.^22^ Also recent works have focused on performing accurate reconstruction methods with limited angle photoacoustic data,^41^ evaluation of binary tomography reconstruction with limited angle data will be taken up as future work. Importantly, our work has focused on two-dimensional image reconstruction of vascular networks; extending the same to three-dimensional geometry should be straight forward as the proposed approach relies on optimization framework (extending the same will be similar to other three-dimensional inversion schemes^33^^,^^42^). Finally, the proposed approach can also be extended to image vasculature networks at mesoscopic depths wherein photoacoustic imaging demonstrated many clinical and preclinical applications.^43^

## 4 Conclusion

The proposed binary PAT method is capable of providing accurate reconstruction of vascular networks directly from the photoacoustic sinogram data. Binary tomography approach relies on solving a dual optimization problem, wherein the primal solution is defined based on a heavy-side function. Further, the dual optimization problem was solved using proximal gradient descent approach with proximal operator being asymmetric soft thresholding. Notably, the reconstructed solution will have a binary distribution based on the sign of the auxiliary variable introduced during the optimization. The binary tomography algorithm was compared with backprojection, Tikhonov regularization, and L1-norm-based regularization approach with *in-silico*, physical phantom, and *in-vivo* brain vasculature data. The results showed that the proposed approach was able to produce results that were closer to the ground truth and the same was reflective from the Dice similarity coefficient. Binary tomography approach could be potentially useful for many applications in the context of photoacoustic microscopy, mesoscopy, and tomography.

## Code, Data, and Materials Availability

The associate code and data that support the findings of this study are available from the corresponding author upon reasonable request.
